# Meat Quality Parameters of Boschveld Indigenous Chickens as Influenced by Dietary Yellow Mealworm Meal

**DOI:** 10.3390/foods10123094

**Published:** 2021-12-14

**Authors:** Letlhogonolo Selaledi, Josephine Baloyi, Christian Mbajiorgu, Amenda Nthabiseng Sebola, Henriette de Kock, Monnye Mabelebele

**Affiliations:** 1 Department of Agriculture and Animal Health, College of Agriculture and Environmental Science, University of South Africa, Florida Campus, 28 Pioneer Ave, Florida Park, Roodepoort 1709, South Africa; laselaledi@zoology.up.ac.za (L.S.); mbajica@unisa.ac.za (C.M.); nasebola@yahoo.com (A.N.S.); 2 Department of Consumer and Food Sciences, Faculty of Natural and Agricultural Sciences, University of Pretoria, Pretoria 0002, South Africa; josephine.baloyi@up.ac.za (J.B.); riette.dekock@up.ac.za (H.d.K.)

**Keywords:** tenebrio molitor, local chicken, carcass characteristics, breast pH, edible insects

## Abstract

An experiment was conducted to examine the effects of yellow mealworm larvae (*Tenebrio molitor*) meal inclusion in diets of indigenous chickens. A total of 160 mixed-sex indigenous Boschveld chickens were randomly divided into four categories: control soybean meal (SBM) and yellow mealworm with percentage levels of 5, 10 and 15 (TM5, TM10 and TM15, respectively). Five replicate pens per treatment were used, with eight birds per pen/replicate. On day 60, two birds from each replicate were slaughtered and eviscerated. Meat quality parameters were measured out on raw carcass and cooked breast meat. The carcass weight, breast weight and gizzard weight of the control group was higher (*p* < 0.05) than the treatment group (TM15). The cooking loss was lower (*p* < 0.05) in the SBM control group but higher in the TM15 group. Colour characteristics of breast meat before cooking was lighter in the TM10 and TM15 group, ranged from 61.7 to 69.3 for L* and was significant (*p* < 0.05). The TM10 and TM15 groups showed a lighter colour than the SBM and TM5 groups. The breast meat pH taken after slaughter was different (*p* < 0.05) in TM5 and TM15, with the highest reading (pH 6.0) in the TM5 group. In conclusion, our experiment indicated that dietary *Tenebrio molitor* in growing Boschveld indigenous chickens’ diets could be considered a promising protein source for Boschveld indigenous chickens.

## 1. Introduction

Smallholder farmers in Southern Africa and many parts of the developing world generally rely on chicken meat to meet their dietary protein requirements [1,2]. The majority of these farmers breeds and keep indigenous chickens, which are mostly tolerant of local diseases and parasites; moreover, they provide huge economic benefits and income for rural communities [3,4]. The sudden increase in the demand for natural or organic meat could have influenced smallholder farmers to consider farming with indigenous chickens because these chickens need minimal use of additives and chemicals [1]. The indigenous chickens are known to be economically, socially and culturally important to the people of Africa and other developing countries, especially those from poor communities [5]. Although they are associated with poor productivity, most consumers prefer their flavoursome meat [5]. However, conventional protein sources, such as soybean and fishmeal, which are normally used in the poultry diets, are expensive, and thus, they lead to the search for alternative sources of protein [6].

Attempts to study alternative sources of protein have included insect meals. Insects are promising animal feed ingredients because they contain high levels of quality protein and are easy to produce [6]. Experimental results have shown that yellow mealworm used as a source of protein in the diets of fast-growing commercial broilers does not compromise growth performance [7,8]. However, there is evidence that indigenous chickens and commercial broiler have genetic differences that could affect growth rates [9]. The strain of the chicken affects the growth rate, feed conversion ratio, feed intake, and digestibility at different ages [9]. In Southern Africa, indigenous breeds, such as Boschveld chicken, which is a dual-purpose crossbreed of three indigenous breed, namely, Ovambo, Matabele and Venda, is normally produced for egg and meat [10]. It is not known how Boschveld indigenous chicken and their physico-chemical parameters could respond to the inclusion of yellow mealworms as a partial replacement of soybean in diets. Biasato et al. [11] studied the effect of yellow mealworm on female fast-growing broilers (Ross 708) and found that carcass weight increased quadratically with increasing levels of yellow mealworms. Furthermore, the abdominal fat weight showed linear responses to increasing levels of yellow mealworms; however, no significant effects were observed from other carcass traits. Cullere et al. [12] studied the effects of insect meal (Black soldier fly) larvae in fast-growing broilers’ (Ross 708) finisher diet on the quality of meat and reported that breast meat displayed similar weight, thawing loss and pH.

To determine the acceptance of chicken meat, consumers consider several characteristics, such as its colour, chemical properties and sensory characteristics [1]. Chicken meat is one of the most consumed in the world due to a number of reasons, which are related to socio-economic conditions, digestion, nutritional value and ease of cooking [13]. Quantifiable properties of meat, such as cooking loss, pH, water holding capacity, protein solubility, fat binding capacity and drip loss, are the most important factors that influence final quality judgement by consumers [14]. Factors such as appearance, colour, tenderness, juiciness and flavour can lead the consumer to approve or not approve the meat product [14]. Therefore, the replacement of soybean meal with yellow mealworms (*Tenebrio molitor*) has the potential to have some effect on the cooking loss, shear force, meat colour and pH of fast-growing broiler chickens [7]. However, there is little information on the use of yellow mealworm meal in the diets of indigenous chickens which are genetically different from fast-growing broilers and their influence on the meat characteristics. Moreover, since the inclusion of yellow mealworms may interfere with the meat quality, studies that go beyond the examination of animal performance are needed. In most cases, the carcass traits of fast-growing broilers are not affected by the inclusion of yellow mealworm in poultry diet [15,16]. However, no studies are available in the literature on the effect of yellow mealworm on carcass traits and meat parameters of slow-growing Boschveld indigenous chickens fed yellow mealworms. Therefore, the aim of this research was to study the effect of yellow mealworm inclusion on the carcass traits and meat quality of Boschveld indigenous chickens.

## 2. Materials and Methods

The present study was conducted at the Proefplaas experimental farm of the University of Pretoria, South Africa. The chickens were reared in a climate-controlled poultry house. The general care and management of the chickens followed accepted guidelines as described by the South African Poultry Association. Furthermore, the experimental protocol was approved by the Human Research Ethics Committee of the College of Agriculture and Environmental Sciences at the University of South Africa with ethical clearance number: 2019/CAES_HREC and the University of Pretoria, Faculty of Veterinary Science, Animal Ethics committee, reference no.: NAS433/2019. The statistical report of the manuscript is added as Supplementary Materials.

### 2.1. Birds and Management

A total of 160-day-old mixed-sex chicks (Boschveld Indigenous chickens) were divided into four equal groups of five replicates (eight birds per replicate). The birds had free access to water and feed and the brooding house was provided with fresh wood shavings as a bedding material. The total number of cages were 20; each cage was 2.5 m × 1 m. The chicks were brooded at 34 °C for a period of one week; thereafter, the temperature was gradually decreased to reach 23 °C. The infrared heat lamps (China Original manufacturer) were used to provide the supplementary heat and maintain stable housing temperature. The housing temperature and relative humidity was monitored continuously, and relative humidity was adjusted to 55. Chicks were vaccinated against Newcastle and Gumboro disease [17]. The chickens were kept in cages for a period of 60 days before slaughter.

### 2.2. Yellow Mealworm Larval Meal and Experimental Diets, Dietary Treatment

A preliminary proximate analysis of oven-dried yellow mealworm was conducted. The oven-dried yellow mealworm nutrient composition is presented in Table 1. Diets were formulated to meet growing indigenous chickens’ requirements. A diet based on corn meal, wheat offal, full-fat soya, sunflower cake and fishmeal was formulated in a feed mill unit in crumble form and served as control. The dietary treatments were as follows: a control group containing 0% yellow mealworm (SBM), TM5, which had 5% inclusion of *tenebrio molitor* larvae, TM10, which had 10% inclusion, and TM15, which had 15% inclusion. The yellow mealworm larvae (20 kg) were collected from the breeding facility of the University of Pretoria, Department of Zoology and Entomology. The other 10 kg was purchased from the local supplier (Insectivore). The yellow mealworm was produced using wheat bran and carrots. Detailed information about the chemical composition of the mealworm is provided in the study by Selaledi and Mabelebele [18]. The yellow mealworms were kept in a −20 °C freezer and were subsequently oven-dried. Two EcoTherm ovens were used to dry the mealworms. A total of 1000 g of mealworm was oven-dried for an hour under a temperature of 120 °C. The experimental diets were isonitrogenous and isoenergetic and were formulated. The diets met or exceeded requirements and were adjusted according to NRC [19].

### 2.3. Slaughter and Carcass Traits (Internal Parts)

On day 60, two birds were randomly selected from each pen (male and female) and transported to the University of Pretoria poultry abattoir, where they were humanely slaughtered. The total number of birds slaughtered was 40; birds were transported in live chicken crates with a lid. The dimensions of each crate were 74 × 53 × 31 cm, with five chickens per crate. Each treatment had two crates, with 5 males and 5 females per treatment. The distance from the poultry house to the abattoir was less than a one kilometre. Birds were transported in a safe reliable vehicle. The birds were stunned using a small electric stunner to induce immediate unconsciousness, which was then followed by severing the neck. After stunning, decapitation and bleeding, the carcasses were plucked, eviscerated and their feet were removed [20]. Carcass weight, eviscerated weight and breast, thigh and drumstick weight were recorded. Indexes of Slaughter rate were calculated as carcass weight/live weight × 100, Eviscerated weight/live weight × 100, Thigh muscle/live weight × 100, Breast muscle/live weight × 100 and wing muscle/live weight × 100 [21].

Immediately after slaughter, the carcasses were labelled and packed in transparent plastic bags and were then kept at 4 °C for 48 h. The carcass was immediately weighed USING THE Mettler PC 4400 SCALE 9Mettler-Toledo, Switzerland) to determine the carcass weight. The carcass was chilled for 48 h and the internal organs were collected and weighed separately. Breast and small intestine (Jejunum) pH was taken immediately after slaughter, followed by another reading after 24 and 48 h, respectively. The pH of the breast and small intestine was measured using a calibrated (standard buffers pH 4.0 and 7.0 at 25 °C) Portable HANNA HI 8424 to measure the pH. The pH meter was calibrated before the first measurement and every time we measured a new set of samples (for every treatment group). The electrode was placed in an incision that was made in the centre of the breast muscle, and for the small intestine, the electrode was placed in the jejunum.

### 2.4. Cooking Procedure

Frozen whole chickens (−20 °C) were thawed overnight at refrigeration temperatures (5 °C) before analysis. After thawing, chickens were placed in individual oven bags Glad^®^ (Clorox Africa) that were tied at one end with a perforated strip. Holes were poked on the oven bag with chicken prior to placing it in the oven, to prevent the bag from bursting in the oven. Bagged chickens were placed in oven pans and cooked in a pre-heated oven at 180 °C for 24 min using Ambassade C505 CT Electric convection oven (Lacanche, France). After cooking and before cooling, the internal temperature of chicken was measured using the Testo 104-IR digital thermometer (Testo SE & Co. KGaA, Lenzkirch, Germany) by inserting the needle probe into the cranial side of both chicken breasts. After cooling, chickens were removed from their bags and weighed. Colour measurements were recorded before the chickens were wrapped in foil and stored in a refrigerator (5 °C) for at least 12 h before texture analysis.

To determine the cooking loss per sample, chickens were weighed before and after cooking. The following formula was used cook loss (%) = ([raw chicken weight–cooked chicken weight]/raw chicken weight) × 100. Cooking loss was calculated for eight chickens, as formula (1) [22]:

Cooking Loss % = (raw chicken weight − cooked chicken weight)/(raw chicken weight) × 100(1)

### 2.5. Texture Properties of Chicken

Chickens were taken out of the refrigerator and left to equilibrate at room temperature (23.0 °C ± 2 °C) for one hour. For each whole chicken, two chicken breasts from the pectoralis major section were manually trimmed using a sharp knife. From each of the cooked chicken breast, one strip (section B), as presented by Lyon and Lyon [22], was cut parallel to the direction of the muscle fibres and used as a test sample. The length, width and thickness of the samples were measured by a Vernier calliper. A shear force test of chicken breasts was performed at room temperature using the texture analyser (Model EZ-L, Shimadzu Tokyo, Japan), with a 5000 N load cell and at a test speed of 50 mm/min. A vertical force was applied on each sample using a Warner-Bratzler shear blade, at an angle perpendicular to the direction of the muscle fibre.

### 2.6. Instrumental Colour Analysis 

The colour of raw and cooked chickens was measured using a CR 210 Minolta chromameter model CR-400 (Osaka, Japan), and an 8 mm aperture size, illuminant D65 and 10° standard observer angle was used. The chromameter was calibrated using a standard white plate (Y = 87.2, X = 0.3173; Y = 0.3348). The colour was measured on breast muscle with three measurements on each sample and an average of three readings per sample was taken. Readings were recorded as L*, a* and b* for four chickens for each treatment. L* represents lightness, a* represents redness (+) and greenness (−) and b* represents blueness (+) and yellowness (−), respectively. Colour measurements were determined for four chickens per treatment [23].

### 2.7. Statistical Analysis

Data were subjected to one-way analysis of variance procedures appropriate for a completely randomised design of IBM SPSS [24]. Means were compared using Tukey’s test. The statement of statistical significance was based on *p* < 0.05. The following model was used for the analysis: Yij = µ + αj + εij, where Yij = Carcass and internal organ variables, µ = the population means, αj = Treatments and εij = the residual effect. Furthermore, the G-Power analysis was used to calculate the statistical power.

## 3. Results

The nutrient composition of the yellow mealworm used in this study and the ingredients used in the experimental diets is presented in Table 1. The carcass characteristics of the 60-day old mixed-sex Boschveld indigenous chickens are shown in Table 2. No significant difference between male and female birds was observed in statistical test. No significant interaction was detected for Eviscerated weight, bled weight, thigh, drumstick, neck and wing, but a significant effect was observed for the carcass weight (Control and TM15), back and breast weight.

The visceral weight, including the head and feet, of 60-day old mixed-sex Boschveld indigenous chickens is presented in Table 3. The dietary treatment (*p* < 0.05) affected the weight of the gizzard in favour of the control group; however, there were no differences between the treatment groups (TM5, 10 15).

**Figure 1:**
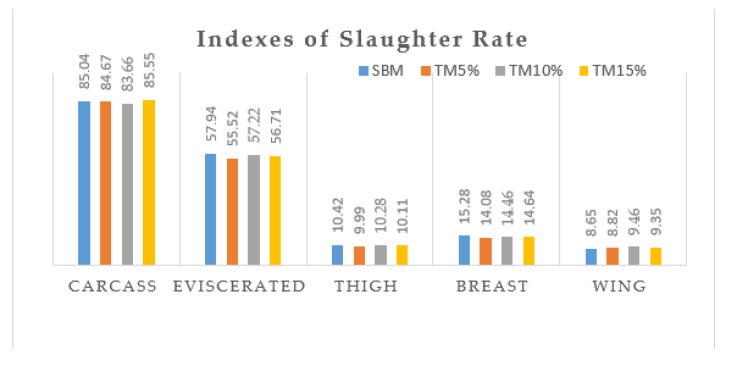
Indexes of slaughter rate of Boschveld chickens fed yellow mealworm.

The slaughter rate is one of many factors affecting the value of a slaughter animal. The indexes of slaughter rate are presented in Figure 1 and were calculated by dividing the weight of carcass by live weight and multiply by 100. The carcass rate was higher in the TM15 group followed by control group (SBM). There were slight variations in the percentages of other meat potions.

Indexes of Slaughter rate were calculated as carcass weight/live weight × 100; Eviscerated weight/live weight × 100; Thigh muscle/live weight × 100; Breast muscle/live weight × 100 and wing muscle/live weight × 100.

Raw and cooked mixed-sex Boschveld indigenous chickens’ meat quality parameters are presented in Table 4. The soybean fed group (SBM) were heavier (*p* < 0.05) than the experimental group (TM15). The cooking loss was lower in the SBM control group as compared to TM15 (*p* < 0.05). However, no differences existed between control, TM5 and TM10 group. The internal temperature was measured immediately after removing the breast meat from the oven. The breast (both left and right) internal temperature was higher in the TM15% group and lower in the SBM group. Colour characteristics was also measured before and after cooking. The values for lightness (before cooking) ranged from 61.7 to 69.3 and this was significant (*p* < 0.05). The TM10 and TM15 group showed lighter colours than the SBM and TM5 groups. No significant (*p* > 0.05) differences were observed for the a*, b* values; similarly, the colour of the breast meat after cooking did not differ in lightness, redness and yellowness. The breast meat from the TM5 group had a lower shear force value (33.7) than the other groups. The TM10 group had the highest shear force (55.6) and stress (0.0927).

Table 5 presents pH values taken 15 min after slaughter followed by another reading after 24 h and 48 h respectively. The breast meat pH taken after slaughter was different (*p* < 0.05) between the TM5 and TM15 groups, with the highest reading (pH 6.0) in the TM5 group. The small intestine pH taken from the jejunum after 24 h was the highest in the SBM group (*p* < 0.05).

## 4. Discussion

Understanding factors that affect chicken meat quality and carcass characteristics are important in the poultry sector [21]. Unfortunately, knowledge on such factors is insufficient, particularly in the indigenous chicken production system. The success of the poultry production enterprise depends on the feed quality given to the chickens [25]. In this study, the inclusion of yellow mealworm meal did not significantly affect the carcass characteristics of the chicken, except on the back and breast meat including the head and gizzard. Similarly, Sedgh-Gooya et al. [15] reported that the carcass traits of broiler chickens fed yellow mealworm larvae powder as a dietary protein source did not significantly affect the carcass characteristics. The study reported that the carcass characteristics such as weight and length of different parts were not influenced by diets that had mealworm powder. Similar results were reported by Bovera et al. [16], which showed that the carcass traits of broilers fed yellow mealworm meal had no significant effect on the carcass traits. However, Zadeh et al. [26] reported a higher carcass yield on the control group of Japanese quails at 35 days of age that were fed yellow mealworm larvae as an alternative protein source. The control group carcass yield for the study by Zadeh et al. [26] was 71.78 higher than TM7.5 (67.42), TM15 (64.47) and TM22.5 (62.24).

With respect to cooking loss, there were no differences among the breast meat between the control, TM5 and TM10 groups. However, the cooking loss in the control group was significantly lower as compared to the TM15 group. These results were consistent with those of Bovera et al. [16], who studied the use of yellow mealworm on broilers. Regarding Bovera’s [16] findings, the cooking loss in the SBM group (21.4) was lower as compared to the TML group (23.6) at 62 d of age. Similarly, Mbhele et al. [27] reported a higher cooking loss in the treatment group (Black soldier fly), although the differences were not significant. Furthermore, Leiber et al. [28] also had a higher cooking loss in the insect fed group (*Hermetia* meal) as compared to the control. Cooking Loss is a significant indicator of meat quality, as it determines the technological yield of the cooking process [29]. Cooking loss is normally calculated as the per cent weight difference between fresh and cooked samples with respect to the weight of fresh meat samples [30]. Therefore, the difference in the cooking loss of the control group that was fed soybean basal diet and TM15 (*Tenebrio molitor*) could suggest that the inclusion of Yellow Mealworm on Boschveld indigenous chickens could have an effect on meat quality parameter (Cooking loss). This result suggests that TM5 and TM10 could be a better inclusion rate of yellow mealworm in Boschveld indigenous chickens’ diet, as there were no significant differences between these treatment groups and the control.

Breast meat from Boschveld chickens that were fed 5% yellow mealworm had a slightly higher average ultimate pH (*p* < 0.05) than the TM15 group; however, the pH from the SBM group was lower than the TM5 group, but the differences were not significant. This reveals that feeding Boschveld indigenous chickens 5% or less of yellow mealworm could cause the breast meat to be less acidic, consumers could prefer meat with pH 5.96 [31]. The breast meat pH values obtained in this study are comparable to those reported by Nhlane et al. [32], who fed Boschveld chickens dietary treatment supplemented with seaweed. These results agree with earlier studies on broilers fed yellow mealworm [16], wherein the TML group (6.12) had the highest pH as compared to the SBM group (5.95). Shaviklo et al. reported that dietary inclusion of mealworm meal up to 3% may be appropriate; however, the meat quality of broilers may adversely be influenced in higher levels [33].

Meat colour can be influenced by environmental and genetic factors [34]. In this study, no difference existed (*p* > 0.05) after cooking among breast meat samples that were harvested from the control and treatment groups. A study conducted by Zadeh et al. [26] on 35-day-old Japanese quails also did not find any significant difference in the breast meat colour (L*), even though their values were lower (43.09–43.41) as compared to this study. The absence of differences in the meat colour after cooking is very important because colour can influence the consumer acceptance of meat (Bovera et al. [16]. The L*a*b* values for all treatments are comparable with the characteristics of normal indigenous chickens’ breast meat; normal broiler meat is described as meat with L* values between 50 and 56, where dark meat will have an L* value <50 and pale meat will have an L* value >56 [35,36]. However, the meat samples from chickens that were fed 10–15% of yellow mealworm larvae were slightly lighter or paler before cooking (68 to 69) than the control group. Lighter breast meat colour is more preferred by consumers than dark meat [34]. Compounds such as myoglobin contribute to the colour of poultry meat [34]. Our L* a*b* findings are not similar to what Bovera et al. [16] reported; the lightness on the breast meat was (44–44.2), redness was (1.07–1.18), yellowness was (0.69–0.78) and chroma was (1.91–1.92). Furthermore, Schiavone et al. [37] did not find differences in the pH and lightness the breast meat between the control and the treatment groups that were fed Black soldier fly as a partial or total replacement of soybean oil.

Additionally, the findings of Bovera et al. [16] of the cooked breast meat shear force was 69.3 for the Soybean group and 73.2 *Tenebrio molitor* group. The shear force from our study was also higher (55.6) among the *Tenebrio molitor* group (TM 10%) but lower than the values reported by Bovera et al. [16]. Meat tenderness is considered a critical attribute in meat consumption. Therefore, it is important to meet the meat tenderness requirements that consumers demand, as this will result in customer satisfaction [9]. Moreover, Mbhele et al. [27] also reported higher shear force values (7.55) in the treatment group of Jumbo Quails that were fed Black soldier fly meal (BSFL50) as compared to the values recorded in the control group (6.20).

## 5. Conclusions

In conclusion, the present experiment indicated that increasing levels of dietary *Tenebrio molitor* in growing Boschveld indigenous chickens diets up to 10–15% could lighten the breast meat colour. It also could improve the pH of the breast meat (TM5) and meat texture (TM5). The yellow mealworms show potential to replace soymeal in slow growing Boschveld indigenous chickens; however, 15% or more could affect performance. Compared with the soybean control group (TM0), some cooking loss occurred in the TM15 group that was fed 15% yellow mealworm. Therefore, this could suggest that feeding Boschveld indigenous chickens 15% or more of yellow mealworm could be disadvantageous. The effect of insect meal on the cooking loss warrants further investigation, because this study and others have reported a higher cooking loss in the insect-fed group (TM15) as compared to the control group. The study confirms that insects can make a valuable contribution as chicken feed to global food security, as suggested already in 1975 by Meyer-Rochow [38].

## Figures and Tables

**Table 1:** Ingredients and the calculated analysis of yellow mealworm larvae meal and experimental diets [18].

Ingredient	SBM	TM5	TM10	TM15	Yellow Mealworm Larvae (g/100 g)
Maize	50.00	50.00	50.00	50.00	
Wheat offal	8.00	8.00	8.00	8.00	
Yellow Mealworm	0.00	5.00	10.00	15.00	
Full fat soya	23.73	20.00	13.00	6.00	
Sunflower cake	10.00	8.73	10.73	12.73	
Fish meal (72%)	3.00	3.00	3.00	3.00	
Limestone	1.20	1.20	1.20	1.20	
Monocalcium phosphate	1.50	1.50	1.50	1.50	
Salt	0.25	0.25	0.25	0.25	
Sodium Bicarbonate	1.50	1.50	1.50	1.50	
DL methionine	0.25	0.25	0.25	0.25	
L-Threonine	0.12	0.12	0.12	0.12	
Lysine HCL	0.25	0.25	0.25	0.25	
Tryptophan	0.05	0.05	0.05	0.05	
Vit TM Premix	0.05	0.05	0.05	0.05	
Min premix	0.05	0.05	0.05	0.05	
Coccidiostat	0.05	0.05	0.05	0.05	
	100.00	100.00	100.00	100.00	
Calculated analysis					
%CP	20.00	20.10	20.17	20.18	51.51
MEKcal/kg	3197.11	3144.25	3110.68	3177.11	24.63
%EE	16.31	16.24	16.72	16.20	25.73
% CF	18.69	18.06	18.29	18.52	6.11
%Ca	1.70	1.67	1.63	1.60	0.294
Avail P%	0.77	0.75	0.72	0.69	7.48
%Lysine	1.92	1.65	1.31	0.97	3.95
Met + Cys%	1.14	1.02	0.87	0.71	n/a

SMB: soybean meal; TM5, TM10 and TM15: *Tenebrio molitor* at 5, 10 and 15% of soybean protein substitution, respectively. CP = crude protein; EE = ether extract; CF = crude fibre; Ca = calcium; P = phosphorus.

**Table 2:** Effects of yellow mealworm meal on carcass characteristics of Boschveld indigenous chickens.

Parameter	SBM	TM5	TM10	TM15	SEM	*p*-Value
Bled weight (g)	807.8	721.6	708	676.4	19.421	0.094
Carcass weight	571.2 ^a^	495.5 ^ab^	530.8 ^ab^	455.8 ^b^	14.637	0.028
Eviscerated weight (g)	550.4	473.2	484.2	448.4	14.943	0.089
Thigh (g)	99	85.2	87	80	3.028	0.169
Drumstick (g)	89.8	74	76	69.4	15.067	0.185
Back (g)	135.2 ^a^	118 ^ab^	117 ^ab^	107 ^b^	3.546	0.045
Neck (g)	52.2	48.4	47.6	44.2	1.586	0.371
Breast (g)	145.2 ^a^	120 ^b^	122 ^b^	115.8 ^b^	3.670	0.015
Wing (g)	82.2	75.2	80	74	2.394	0.593

^a,b^ Means in the same row not sharing a common superscript are different (*p* < 0.05). Diets: SBM = soybean meal, TM5% = a diet in which 5 g/kg of soybean was replaced with *Tenebrio molitor* meal, TM10% = a diet in which 10 g/kg of soybean was replaced with *Tenebrio molitor* meal. TM15% = a diet in which 15 g/kg of soybean was replaced with *Tenebrio molitor* meal. SEM: standard error of the mean.

**Table 3:** Effects of the percentage of yellow mealworm meal on organ weight characteristics of Boschveld indigenous chickens.

Parameter	SBM	TM5	TM10	TM15	SEM	*p*-Value
Head (g)	34.2 ^a^	29.8 ^ab^	31.2 ^ab^	28.0 ^b^	0.837	0.055
Feet (g)	37.6	31.2	35.6	31.6	1.403	0.304
Gizzard (g)	30.6 ^a^	19.0 ^b^	21.2 ^b^	21.4 ^b^	1.032	0.000
Lungs (g)	6.0	6.0	4.6	5.0	0.269	0.155
Liver (g)	18.4	14.8	16.4	16.2	0.626	0.245
Heart (g)	5.6	5.6	4.4	5.2	0.212	0.148
Proventriculus (g)	4.2	3.6	4.8	4.2	0.245	0.408
Small intestine (g)	20.4	18.4	17.8	17.2	0.570	0.220
Full intestine length (cm)	117.5	119.7	116.4	111.5	2.175	0.609

^a,b^ Means in the same row not sharing a common superscript are different (*p* < 0.05). Diets: SBM = soybean meal, TM5% = a diet in which 5 g/kg of soybean was replaced with *Tenebrio molitor* meal, TM10% = a diet in which 10 g/kg of soybean was replaced with *Tenebrio molitor* meal. TM15% = a diet in which 15 g/kg of soybean was replaced with *Tenebrio molitor* meal. SEM: standard error of the mean.

**Table 4:** Physical properties of carcass without internal viscera of Boschveld indigenous chickens fed soybean meal (SBM) or *Tenebrio molitor* larvae meal (TML) at 60 day of age.

Parameters (Before Cooking)	SBM	TM5%	TM10%	TM15%	SEM	*p*-Value
Breast meat before cooking						
Lightness L* (before cooking)	61.8 ^b^	61.7 ^b^	69.3 ^a^	68.8 ^a^	1.201	0.008
Redness a* (before cooking)	1.9	1.7	3.0	2.7	0.354	0.549
Yellowness b* (before cooking)	2.9	6.3	5.0	5.0	0.651	0.352
Parameters (After cooking)						
Cooking loss %	8.82 ^b^	12.23 ^ab^	11.97 ^ab^	13.70 ^a^	0.650	0.049
Breast Internal temperature (left)	65.3 ^b^	76.7 ^a^	71.3 ^ab^	80.7 ^a^	1.702	0.004
Breast Internal temperature (right)	66.0 ^b^	76.5 ^a^	72.4 ^ab^	80.8 ^a^	1.570	0.003
Drained water (g)	29.0	33.1	34.3	36.4	1.516	0.388
Lightness L* (after cooking)	71.4	66.4	69.4	68.8	0.950	0.343
Redness a* (after cooking)	3.8	4.3	2.8	2.8	0.259	0.076
Yellowness b* (after cooking)	17.0	21.1	17.2	17.6	0.661	0.077
Texture (shear force)	40.9 ^b^	33.7 ^b^	55.6 ^a^	39.8 ^b^	2.166	0.001
Texture (stress)	0.0527 ^b^	0.0574 ^b^	0.0927 ^a^	0.0682 ^ab^	0.004	0.003

^a,b^ Means in the same row not sharing a common superscript are different (*p* < 0.05). Diets: SBM = soybean meal, TM5% = a diet in which 5 g/kg of soybean was replaced with *Tenebrio molitor* meal, TM10% = a diet in which 10 g/kg of soybean was replaced with *Tenebrio molitor* meal. TM15% = a diet in which 15 g/kg of soybean was replaced with *Tenebrio molitor* meal. SEM: standard error of the mean.

**Table 5:** The pH values of breast and small intestine taken from 60-day-old mixed-sex Boschveld indigenous chickens.

Parameter	SBM	TM5%	TM10%	TM15%	SEM	*p*-Value
Breast pH (After slaughter)	5.83 ^ab^	6.01 ^a^	5.80 ^ab^	5.74 ^b^	0.037	0.055
Breast pH (After 24 h)	5.88 ^a^	5.87 ^ab^	5.80 ^ab^	5.75 ^b^	0.017	0.034
Breast pH (After 48 h)	5.86	6.02	5.86	5.98	0.028	0.093
Small intestine pH (After slaughter)	6.37	6.18	6.19	6.20	0.031	0.093
Small intestine pH (after 24 h)	6.45 ^a^	6.23 ^b^	6.29 ^b^	6.30 ^ab^	0.023	0.003
Small intestine pH (after 48 h)	6.28	6.17	6.20	6.27	0.020	0.174

^a,b^ Means in the same row not sharing a common superscript are different (*p* < 0.05). Diets: SBM = soybean meal, TM5% = a diet in which 5 g/kg of soybean was replaced with *Tenebrio molitor* meal, TM10% = a diet in which 10 g/kg of soybean was replaced with *Tenebrio molitor* meal. TM15% = a diet in which 15 g/kg of soybean was replaced with *Tenebrio molitor* meal. SEM: standard error of the mean.

## Data Availability

Data are contained within the article.

## Supplementary Materials

The following are available online at https://www.mdpi.com/article/10.3390/foods10123094/s1.
