# Supplementary material for: Meat Quality Parameters of Boschveld Indigenous Chickens as Influenced by Dietary Yellow Mealworm Meal
Source: Foods. 2021 Dec 14;10(12):3094. doi: 10.3390/foods10123094 (PMC8701880; doi:10.3390/foods10123094)
Supplement: Supplementary file 1 [file foods-10-03094-s001.zip › foods-1430295-supplementary.pdf]

Statistical report of the manuscript submitted for publication

Manuscript title: Meat Quality Parameters of Boschveld Indigenous Chickens as Influenced by Dietary Yellow Mealworm Meal

Authors: Letlhogonolo Selaledi, Josephine Baloyi, Christian Mbajiorgu, Amenda Nthabiseng Sebola, Riette De Kock and Monnye Mabelebele

Journal: Foods

Biometrician comments

**Statistical analysis section**	Data was subjected to analysis of variance procedures appropriate for a completely randomised design and analysed by one-way ANOVA using the general linear model procedures of IBM SPSS [19]. Means were compared using the Duncan multiple range test. Statement of statistical significance is based on p<0.05.
**Statistical analysis suggested**	Data was subjected to one-way analysis of variance procedures appropriate for a completely randomised design of IBM SPSS [19]. Means were compared using the Tukey's test. Statement of statistical significance is based on p<0.05. The following model was used for analysis: $Yij = \mu + \alpha j + \varepsilon ij$<br>Where; Yij was ………, μ was ………, αj was………and εij was……<br>"The question of a model (fixed and random effects) will be answered by adding this model"
**Mean separation procedure used**	Duncan multiple range test
**Mean separation procedure suggested**	Tukey test
**Overall comments**	The statistical technique used was one-way ANOVA not with general linear model because the number of observations were equal in all the treatments, hence I suggested the authors remove the general linear model procedure on their analysis section. The number of treatments are only 4 (SBM, TM5, TM10, TM15) and one of the treatment is the control (SBM). Therefore, Duncan multiple range test does not qualify to compare the treatment means, hence I suggested the Tukey test. All these statistical errors affected the results hence the tables are questionable. For example, on Table 2 the p-value of Eviscerated weight is 0.089 which is not significant but Duncan multiple range test used showed it significant. Replace Duncan multiple range test with Tukey test to get statistically reasonable results.

From Dr TL Tyasi (Biometrician)

Doctor Thobela Louis Tyasi
Applied Statistics Independent Consultant
Biometrician/Biostatistician

09 NOV 2021

BSc. Hons; MSc; PhD;
Statistical Programming (Licence no. BP05380v9)
E-mail: tyasi1921@gmail.com
